# Association between hydroxychloroquine intake and damage to the outer nuclear layer in eyes without manifest retinal toxicity

**DOI:** 10.1186/s12886-024-03684-3

**Published:** 2024-09-27

**Authors:** Nagib Salameh, Carla Abi Doumit, Edmond Jalkh, Joseph Nehme

**Affiliations:** 1 Eye and Ear Hospital International, Naccache, Lebanon; 2 Faculty of Medicine, Holy Spirit University of Kaslik (USEK), Jounieh, Lebanon

**Keywords:** Hydroxychloroquine, Outer nuclear layer thinning, Early detection of retinopathy, Autoimmune diseases

## Abstract

**Background:**

Hydroxychloroquine (HCQ) is widely used to treat various autoimmune diseases but carries a risk of retinal toxicity, particularly with prolonged use. Despite advancements, uncertainty persists regarding optimal screening methods. Recent advances in OCT have enabled early detection of retinal damage, with studies suggesting that thinning of specific retinal layers may be an early indicator of toxicity. However, there is a gap in research on outer nuclear layer (ONL) thinning in HCQ users without apparent retinal toxicity. This information is crucial for improving screening and identifying the ONL as a reliable biomarker for screening. Therefore, this study aimed to investigate the association between HCQ intake and ONL damage in eyes without manifest retinal toxicity.

**Methods:**

A case‒control study was conducted at the ophthalmology department of Eye and Ear Hospital International from July 2022 to June 2023. The study included 20 individuals on HCQ and 20 age-matched controls. The data were obtained through chart reviews, and participants underwent comprehensive ophthalmic assessments.

**Results:**

A total of 80 eyes were analyzed. Patients on HCQ exhibited significantly thinner perifoveal, parafoveal, and overall ONL compared to controls (*P* < .001, *P* < .012, and *P* < .004, respectively). Similarly, this association was found in the nasal, inferior, and temporal quadrants of both the inner (region 3: *P* < .01, region 4: *P* < .001, and region 5: *P* < .03) and outer zones (region 7: *P* < .04, region 8: *P* < .001, region 9: *P* < .02), most pronounced in the inferior regions. The cumulative dose was weakly associated with decreased ONL thickness only in the nasal quadrant of the inner zone (region 3: *P* < .047). Correlation analysis of the initial and most recent OCT scans in the same individuals revealed a weak association with ONL thinning in the central zone (region 1: *P* < .0048).

**Conclusion:**

The thickness of the ONL can significantly decrease in patients taking HCQ, even in the absence of of manifest retinal toxicity. This study is the first to evaluate this association in eyes with negative screening and diagnostic tests for HCQ retinopathy. The findings suggest that ONL thickness could serve as an early diagnostic indicator for HCQ retinal toxicity.

## Introduction

For numerous years, the medical community has recognized the potential retinal toxicity linked to chloroquine (CQ) and its analog hydroxychloroquine (HCQ) [1]. Physicians from diverse specialties have integrated HCQ into the treatment of various conditions, such as lupus and rheumatoid arthritis, in addition to exploring broader uses such as diabetes mellitus management [2]. Despite its relatively low cost and favorable safety profile compared to other disease-modifying antirheumatic drugs (DMARDs), retinal toxicity remains a recognized adverse outcome associated with the prolonged use of HCQ [3]. Several factors contribute to an increased risk of retinal toxicity, including extended use (> 5 years), a higher cumulative dose, exceeding the recommended daily dose based on real or ideal body weight, concurrent use of tamoxifen, specific cytochrome P450 gene variations, and preexisting retinal, hepatic, or renal conditions [1]. Prolonged HCQ intake may lead to retinopathy, which is characterized by damage to the parafoveal retina [4]. Cessation of the drug does not arrest the progression of retinopathy or restore vision. Remarkably, patients exhibiting HCQ retinopathy displayed sustained retinal damage on optical coherence tomography (OCT) for more than 3 years following discontinuation of the drug [5].

Moreover, it has been suggested that patients might exhibit varying degrees of sensitivity to different tests resulting in a lack of consensus within the literature concerning the ideal screening test for HCQ toxicity [6]. The foremost screening techniques encompass automated visual fields (VFs) in conjunction with spectral-domain optical coherence tomography (SD-OCT). Objective confirmation of VF can be obtained through multifocal electroretinography (mfERG), whereas fundus autofluorescence (FAF) proves valuable in detecting topographical damage [1].

Although the pathophysiology of HCQ retinopathy remains incompletely understood, significant progress has been made in elucidating this condition through clinical imaging studies [7]. Significant advancements in OCT imaging and resolution have enabled the detection of initial retinal damage before visible fundus changes occur [7]. This capability arises not only from the impressive specificity and objectivity of OCT but also from its widespread accessibility to ophthalmologists [8]. OCT segmentation provides the capability to present precise measurements of retinal layer thickness, facilitating a comprehensive analysis of each individual layer. Recent findings have led some researchers to hypothesize that ganglion cell-inner plexiform layer (GCIPL) thinning could be an early screening indicator [9]. However, recent investigations have revealed hydroxychloroquine-induced damage in the outer retina or choroidal tissue, which is consistent with observations from in vitro studies [10–13]. Additionally, while an association between outer nuclear layer (ONL) thinning and HCQ retinopathy was found among the few studies that were conducted [14–16], there is a notable absence of studies aimed at quantifying the extent of disparity between healthy controls and affected individuals. Moreover, it is important to note that, to date, no study has specifically examined ONL thinning in individuals on HCQ with negative screening and diagnostic tests for HCQ retinopathy. This gap in research is imperative for the effective screening of HCQ retinopathy to determine whether specific measurements can serve as reliable and objective biomarkers for toxicity. Therefore, the aim of this study was to investigate the association between HCQ intake and ONL damage in eyes without manifest retinal toxicity.

## Materials and methods

### Patient population

This retrospective chart review was conducted from July 2022 to June 2023 at Eye and Ear Hospital International, Naccache, Mont-Liban, Lebanon. The research protocol was approved by the ethics committee of Eye and Ear Hospital International. Ethical considerations were addressed as the research was conducted in accordance with the ethical principles of the Declaration of Helsinki.

The study followed a case‒control design. Patients who were taking HCQ were included as cases, while patients in the control group were healthy, not receiving HCQ. The data were acquired from the hospital’s database, and a total of 35 individuals receiving HCQ were identified. The inclusion criterion was individuals who had been using HCQ for a minimum of 5 years, given that screening is initiated annually 5 years after starting treatment [1]. Eligible participants should also exhibit a normal fundus exam, VF, OCT and mERG. The exclusion criteria were prior retinal disorders (such as macular degeneration, central serous chorioretinopathy, or diabetic retinopathy), glaucoma, hepatic or renal insufficiency, treatment with medications associated with retinal toxicity (e.g., tamoxifen) and high myopia (> 6 diopters). Eight people were subsequently excluded based on these criteria. The remaining 27 individuals had no prior OCT, and they were subsequently invited via telephone to undergo this imaging. The recruitment was conducted by a single trained person. Additional assessments of VF and mERG were repeated if the initial results were over 1 year old [1]. Two participants were excluded from the study due to abnormal VF results, one participant was excluded due to abnormal mERG findings, and an additional four patients were excluded because they did not attend the scheduled invitation. Ultimately, a cohort of 20 individuals (18 females and 2 males) with 40 analyzable eyes was included in the study (Table 1). The control group comprised an age- and sex- matched selection of 20 healthy participants not receiving HCQ and with normal OCT images, totaling 40 eyes. Medical records were reviewed for each participant, documenting essential information such as age, sex, diagnosis, associated comorbidities, daily HCQ dosage, cumulative dose, and treatment duration. Furthermore, correlations were detected between the initial and most recent OCTs among individuals who were on HCQ, with the lapse of time between the OCTs being at least 2 years. There were 11 patients who met these criteria, resulting in a total of 22 eyes being included in this aspect of the study.

**Table 1:** Characteristics of participants taking hydroxychloroquine

Patient no.	Eyes	Sex	Age	Diagnosis	Daily dose (mg/d)	Treatment duration (years)	Cumulative dose (g)
1	OU	F	66	Sjogren	400	6	876
2	OU	F	56	SLE	400	16	2336
3	OU	F	58	SLE	400	6	876
4	OU	F	17	SLE	400	9	1314
5	OU	F	58	Sjogren	400	8	1168
6	OU	F	66	RA	400	15	2190
7	OU	F	58	Sjogren	400	9	1314
8	OU	F	60	RA	400	5	730
9	OU	M	50	Sjogren	400	6	876
10	OU	F	63	RA	400	5	730
11	OU	F	33	Scleroderma	200	9	657
12	OU	F	35	Scleroderma	400	5	730
13	OU	F	51	SLE	400	5	730
14	OU	M	46	RA	400	7	1022
15	OU	F	64	SLE	400	20	2920
16	OU	F	29	SLE	400	7	1022
17	OU	F	75	GCA	400	15	2190
18	OU	F	74	SLE	400	6	876
19	OU	F	62	Sjogren	400	5	730
20	OU	F	57	Sjogren	400	7	1022

### Clinical assessment

On the day of OCT imaging, a comprehensive ophthalmic assessment was conducted for each participant by the same retina specialist. This assessment included sequential evaluations with best-corrected visual acuity (BCVA), slit lamp examination, fundus examination, and OCT imaging. VF and mERG data were retrieved from records or repeated as necessary if the time elapsed since the last exam exceeded 1 year [1].

### Visual field

All enrolled patients underwent a 10 − 2 VF assessment using the standard automated perimetry (SAP, Octopus 101, G2). The categorization of the VF as normal was based on the absence of scotoma according to the sector (nasal, temporal, superior, and inferior), the mean deviation (MD) and the loss of variance (LV).

### Optical coherence tomography

**Fig. 1:**
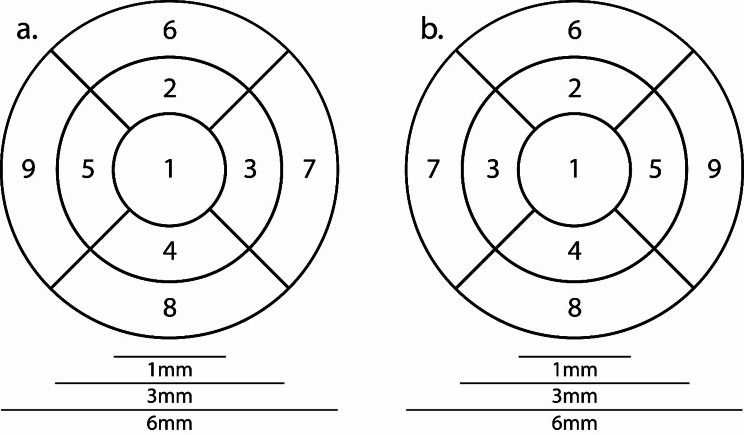
Early Treatment Diabetic Retinopathy Study (ETDRS) grid overlay. The ETDRS grid includes concentric circles of 1, 3, and 6 mm around the fovea, illustrating the nine subfields within each of the nine ETDRS regions. This grid is used to assess and quantify retinal thickness. (**a**) Right eye and (**b**) left eye

OCT measurements were performed by the same ophthalmologist using the Spectralis HRA + OCT machine (Heidelberg Engineering, Heidelberg, Germany) to assess retinal thickness. The Heidelberg Eye Explorer software (version 1.10.12.0) was used for image processing and segmentation. This involved selecting the “segmentation” and “all layers” function by right-clicking on the volume scan in the software, followed by viewing the image under the “Thickness Map” tab with the Early Treatment Diabetic Retinopathy Study (ETDRS) grid overlay, featuring concentric circles of 1, 3, and 6 mm around the fovea (Fig. 1). The ONL was chosen from the “Layers” menu, and was precisely isolated with boundary lines from the bottom of the outer plexiform layer to the external limiting membrane. Verification of boundary accuracy and correct ETDRS overlay alignment was performed by the same ophthalmologist of the research team for each image within the volume scan. Only high-quality scans were included; any poor-quality scans were immediately repeated. Remarkably, no manual adjustments were necessary for the study scans. Isolated ONL measurements were recorded for each of the 9 ETDRS sections as generated by the algorithm (Fig. 1). Overall thickness measurements were obtained by summing the subsections indicated in the ETDRS grid.

### Multifocal electroretinography

mERG was performed following the guidelines set by the International Society for Clinical Electrophysiology of Vision. Using the visual evoked response imaging system (VERIS), multifocal ERG assessments were conducted by an optometrist. They were subsequently reviewed by a single ophthalmologist.

### Statistical analysis

Descriptive and inferential statistics were calculated using SPSS 25. Descriptive statistics involved generating frequency tables, means, standard deviations, and graphs for both the case and control groups. Inferential statistics comprised assessing normality through skewness and kurtosis values. Parametric tests were applied to variables within the acceptable range (-2 to 2), including the t-test for mean comparison and Pearson correlation for bivariate correlations. Nonparametric tests were used for values outside this range, such as the Mann-Whitney Test for mean comparison, Spearman correlation for bivariate correlations, and Wilcoxon Signed Ranks test for comparing old and new values in the case group. A p-value < 0.05 indicated a significant difference, while a p-value > 0.05 indicated a nonsignificant difference.

In addition, Levene’s test was used for variance comparisons between groups. This test was conducted as part of the overall statistical analysis process to ensure the validity of the assumptions, particularly in the context of parametric tests.

## Results

**Fig. 2:**
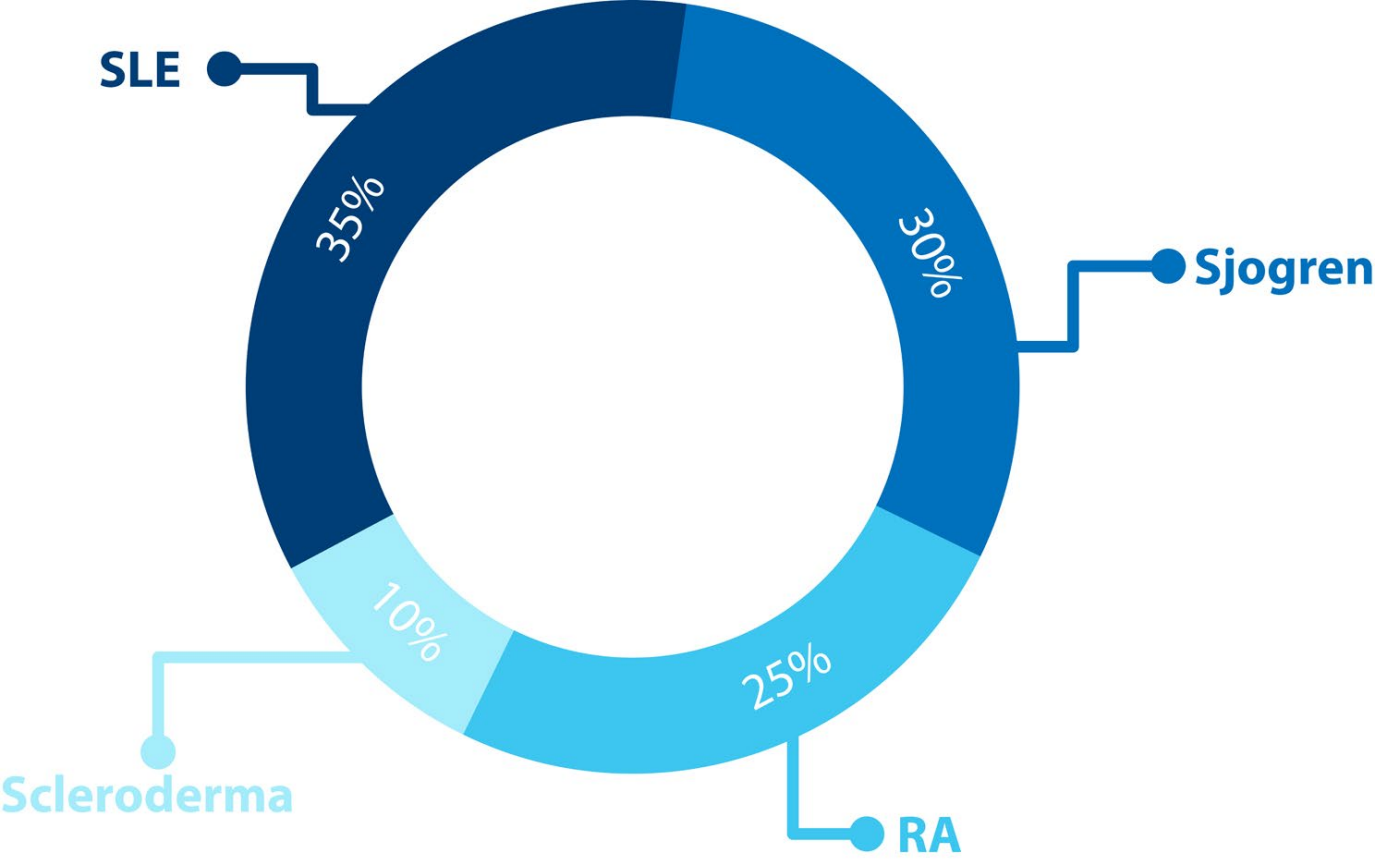
Distribution of autoimmune diseases among patients taking hydroxychloroquine. The pie chart shows that 35% of the patients were treated for systemic lupus erythematosus (SLE), 30% for Sjogren syndrome, 25% for rheumatoid arthritis (RA), and 10% for scleroderma

Overall, 80 eyes of 40 individuals (36 females and 4 males) were included in the study. The case group comprised 20 individuals with a total of 40 eyes. The mean treatment duration was 8.6 ± 4.4 years (range: 5–20 years), the mean age was 55 ± 15 years (range: 33–75 years), and the mean spherical equivalent was 0.56 ± 1.1 diopters. The control group, consisting of 20 individuals with a total of 40 eyes, had a mean age of 58 ± 14 years (range: 26–75 years) and a mean spherical equivalent of 0.36 ± 0.9 diopters. No statistically significant differences in age, sex or spherical equivalent were observed between the groups. Seven individuals (35%) were treated for systemic lupus erythematosus (SLE), 6 (30%) for Sjogren syndrome, 5 (25%) for rheumatoid arthritis (RA), and 2 (10%) for scleroderma (Fig. 2).

**Fig. 3:**
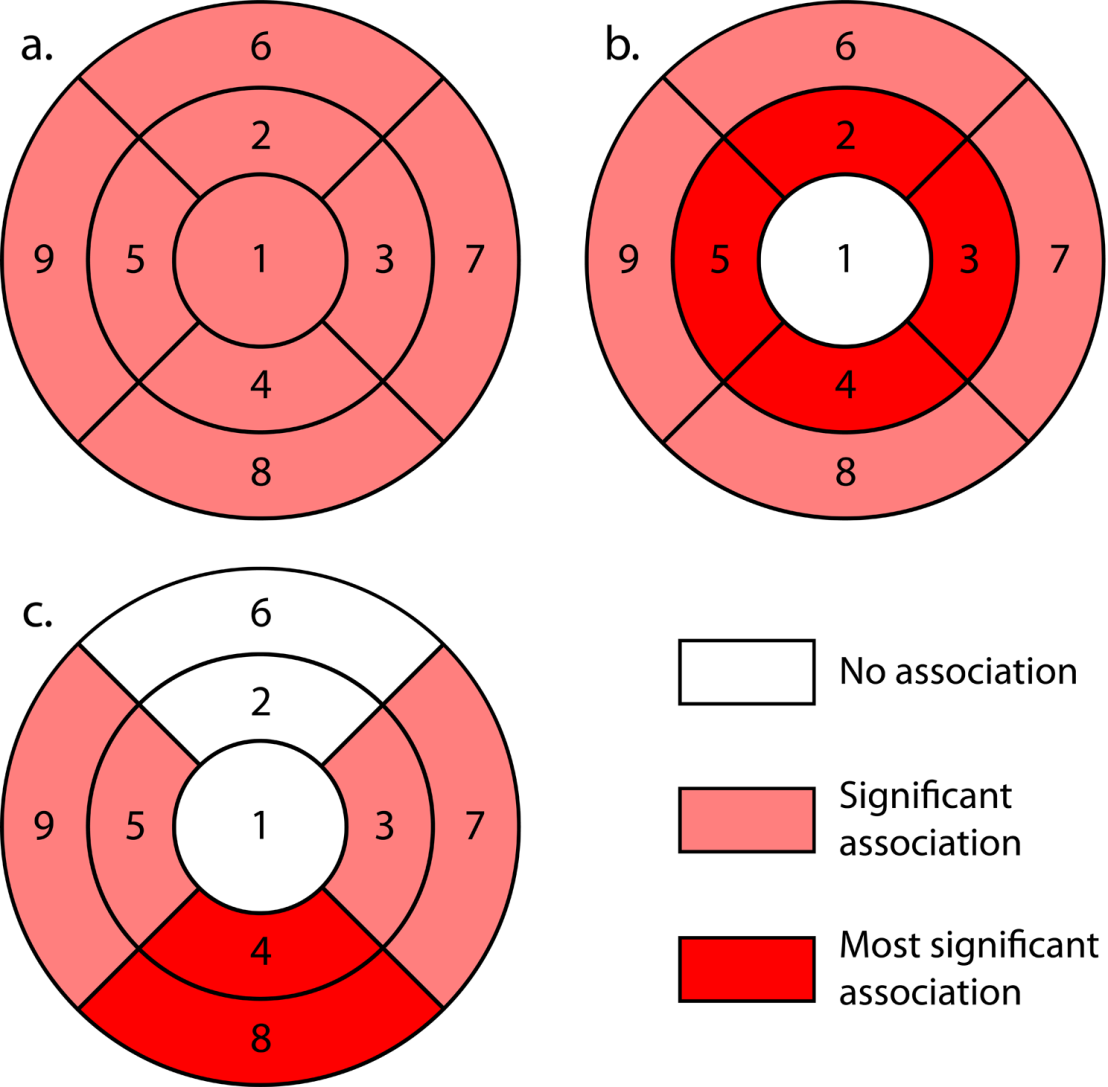
Analysis of outer nuclear layer (ONL) thickness in patients on hydroxychloroquine (HCQ) therapy compared to healthy controls. These are ETDRS grids of the right eye: (**a**) illustrates a significantly thinner overall ONL region in HCQ-treated patients compared to controls (*P* < .004). (**b**) highlights the differences in ONL thickness within the perifoveal and parafoveal regions, showing marked thinning in HCQ patients (*P* < .001 and *P* < .012, respectively), with the most pronounced thinning occurring in the perifoveal region. (**c**) shows the regional variations within the ETDRS grid, with significant thinning observed in the nasal, inferior, and temporal quadrants of both the inner (region 3: *P* < .01, region 4: *P* < .001, and region 5: *P* < .03) and outer zones (region 7: *P* < .04, region 8: *P* < .001, region 9: *P* < .02) most pronounced in the inferior regions

The results from the segmentation data analysis (Table 2) revealed a significantly thinner overall ONL region in patients taking HCQ than in healthy controls (*P* < .004) (Fig. 3a). Thickness measurements comparing the perifoveal and parafoveal regions showed significant differences between patients on HCQ and controls (*P* < .001, and *P* < .012 respectively) resulting in ONL thinning (Fig. 3b). For each individual region in the EDTRS cube, the comparison revealed significant changes in the nasal, inferior, and temporal quadrants of both the inner (*P* < .01, *P* < .001, and *P* < .03 respectively) and outer zones (*P* < .04, *P* < .001, and *P* < .02 respectively) (Fig. 3c). The strongest associations were observed in the inferior regions, particularly in the inferior quadrants of the inner and outer zones. No significant differences in ONL thickness were detected in the central zone and the upper quadrants of both the inner and outer zones (*P* < .26, *P* < .41, and *P* < .73 respectively).

**Table 2:** Comparison of outer nuclear layer thickness between eyes of patients undertaking hydroxychloroquine and control eyes

ONL ETDRS region	Eyes of patients on HCQ (*n* = 40)	Control eyes (*n* = 40)	*p* value
1	83.8	87.9	0.26
2	60.8	63.1	0.41
3	63.5	70.7	0.01
4	56.8	67.4	0.001
5	62.9	69.4	0.03
6	56	56.1	0.73
7	50.5	55.7	0.04
8	46.4	51.6	0.001
9	50.9	56.5	0.02
Pericentral ring*	61	67.6	0.001
Peripheral ring**	50.9	55	0.012
Total	59.1	64.3	0.004

*Pericentral ring: regions 2, 3, 4, and 5; **Peripheral ring: regions 6, 7, 8, and 9

**Fig. 4:**
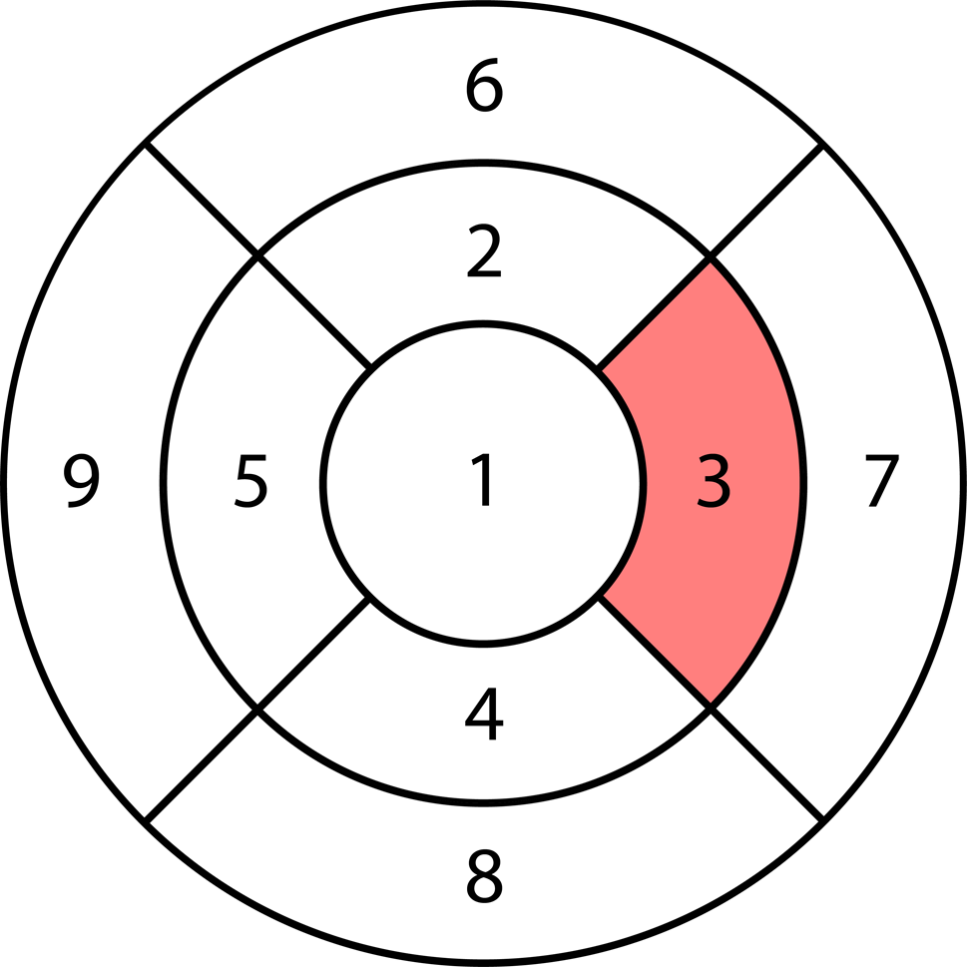
Correlation between HCQ intake and the thickness of the ONL in different retinal regions. This is an ETDRS grid of the right eye. The cumulative dose of HCQ shows a significant decrease in ONL thickness in the nasal quadrant of the inner zone (region 3: *P* < .047)

The correlation test indicated that as the duration of HCQ intake increased, the thickness of the ONL in the different regions tended to decrease, with varying degrees of strength. However, no significant associations were noted. The same pattern was observed for the cumulative dose of HCQ and ONL thickness, except that this association in the nasal quadrant of the inner zone (*P* < .047) was moderately strong and statistically significant (Fig. 4).

**Fig. 5:**
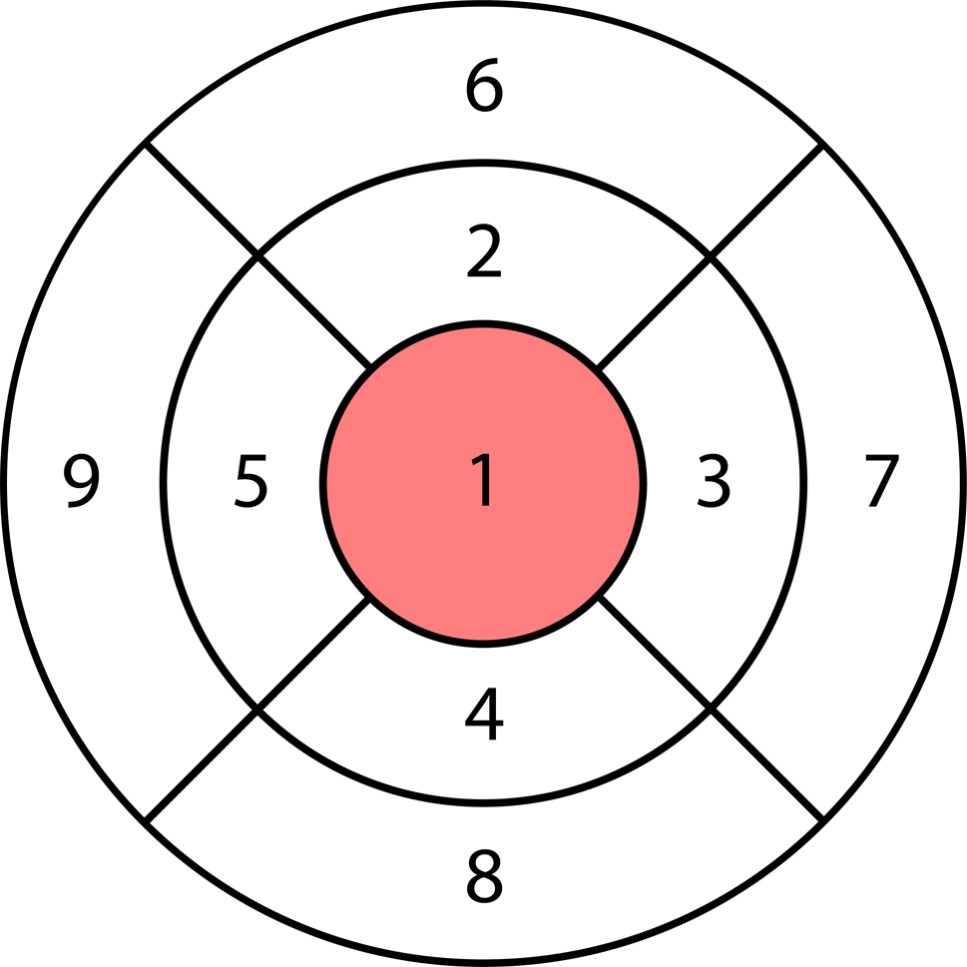
Correlation between initial and most recent OCT findings among individuals who were prescribed HCQ. This is an ETDRS grid of the right eye. A significant association with ONL thinning in the foveal zone was observed (region 1: *P* < .048)

The mean treatment duration on initial OCT, most recent OCT, and the duration difference between the OCT scans were 5.7 ± 3.1 years (range: 3–13 years), 11 ± 4.4 years (range: 6–20 years), and 5.3 ± 1.6 years (range: 2–7 years), respectively (Table 3). The correlation between initial and most recent OCT findings among individuals who were on HCQ revealed a weak significant association with ONL thinning in the central region (*P* < .048). However, no statistically significant correlation was detected between the overall ONLs (*P* < .504) and the remaining of the ETDRS grid regions (region 2: *P* < .0.562, region 3: *P* < .614, region 4: *P* < .0.384, region 5: *P* < .676, region 6: *P* < .694, region 7: *P* < .465, region 8: *P* < .541, region 9: *P* < .549) (Fig. 5).

**Table 3:** Treatment duration comparison: initial OCT, most recent OCT, and duration differences

Patient no.	Duration of treatment on initial OCT	Duration of treatment on most recent OCT	Duration difference between the initial and latest OCT
2	9	16	7
4	4	9	5
5	4	8	4
6	8	15	7
7	3	9	6
11	4	9	5
14	3	7	4
15	13	20	7
17	8	15	7
16	3	7	4
18	4	6	2

## Discussion

HCQ, a widely recognized medication for its efficacy in preventing autoimmune diseases such as RA and SLE, has been extensively studied for its potential side effects. One notable concern is its association with irreversible retinal damage, which leads to both structural and functional progression. Despite its proven effectiveness and cost-effectiveness in managing autoimmune diseases, the evidence underscores the necessity of vigilant monitoring for potential toxicity. Recognizing the importance of early detection, various tests are employed to assess signs of HCQ toxicity, preventing unwarranted discontinuation of the medication. To verify HCQ toxicity and make informed decisions regarding discontinuation, two screening tests are recommended. According to the current guidelines from the American Academy of Ophthalmology (AAO), a combination of VF and SD-OCT is recommended due to its widespread availability. To our knowledge, this research contributes by being the first to evaluate this association in patients taking HCQ with negative screening and diagnostic tests for retinal toxicity, which could be used as a new objective screening test for HCQ retinopathy.

The current study revealed an association between HCQ intake and ONL damage: a reduction in perifoveal, parafoveal, and overall ONL thickness among patients taking HCQ in comparison to the control group was found. Subgroup analyses further supported the observed relationship. For instance, significant alterations in ONL thickness were observed in specific regions of the ETDRS cube, specifically nasal, inferior, and temporal quadrants of the inner and outer zones, with the most pronounced association noted in the inferior perifoveal and parafoveal quadrants. The cumulative dose was weakly associated with decreased ONL thickness only in the nasal quadrant of the inner zone. Furthermore, initial and most recent OCT scans in the same individuals showed a weak association with ONL thinning in the foveal region.

Our findings were consistent with those of prior studies [14–16], indicating that patients taking HCQ exhibited a thinner overall ONL than age-matched healthy controls. However, our study introduces nuances in comparison to the literature. For instance, Casado et al. [15] linked ONL thinning to HCQ retinopathy in high-risk patients by categorizing them based on the presence of HCQ retinopathy. In contrast, our study utilized HCQ intake as a discriminator for the risk of HCQ retinopathy, which puts these patients at greater risk of developing this retinopathy. This makes it potentially more valuable for early disease detection in a screening context, which aims to detect the disease earliest before it manifests.

Our results further revealed associations between HCQ intake and thinning of retinal regions in the temporal, nasal, and inferior segments of both the perifoveal and parafoveal retina, with the most significant associations found in the inferior regions. Additionally, the cumulative dose of HCQ was associated with ONL damage in the perifoveal nasal region. These findings align with some aspects of prior studies, such as those of Casado et al. [15] and Jain et al. [16], but discrepancies exist, particularly in the locations of the most crucial associations. Casado et al. [15] reported a significantly thinned retina in the inferior and nasal retina, whereas Jain et al. [16] found that the temporal and nasal subsections displayed the narrowest range of data in the parafoveal zone, especially in the nasal zone. These results suggest that our findings regarding the association between the cumulative dose and ONL thinning are in concordance with those of previous studies. However, the association between ONL thinning and HCQ intake on OCT scans of cases and controls is discordant, with our study indicating that the inferior regions have the most significant association. The variations in results across studies may be attributed to the limited sample sizes of the different studies.

Interestingly, our study identified a novel aspect regarding the association between initial and most recent OCTs with ONL damage, revealing thinning in the foveal zone. This phenomenon has not been previously described in studies focused on ONL thinning. However, a separate study [17] on chronic exposure to HCQ, linked it to thinning of the macular ganglion cell-inner plexiform layer (GC-IPL), even in the absence of functional or structural clinical changes. This observation supports the hypothesis that retinal anatomical changes may manifest initially in the foveolar area, aligning with the typical presentation of early-stage hydroxychloroquine retinal toxicity characterized by partial paracentral or complete pericentral ring scotomas [18, 19]. Our study contributes to the literature by specifically identifying the macular region and cellular levels in the ONL that undergo selective thinning.

This study has several limitations that warrant consideration. Although the sample size was small, it was deemed sufficient based on strong confidence intervals, suggesting that it was adequate for the study. Despite evaluating patients using VF, OCT, mERG, and fundoscopy, the omission of FAF represents a potential limitation. Furthermore, the exclusion of some patients who missed follow-up OCT appointments and the lack of a confirmed normal range for ONL thickness introduce challenges in the interpretation of the results. An additional limitation is the potential for gender bias, given the cohort composition of 18 females and only 2 males. Moreover, caution is advised in generalizing these results to other populations, as the findings may be influenced by equipment-specific thickness values and ethnic differences.

In conclusion, this study revealed a significant correlation between HCQ intake and ONL thinning in eyes without manifest retinal toxicity. The assessment of these changes was conducted using the ETDRS grid. Remarkably, this study represents a pioneering effort to explore this association in individuals with negative screening and diagnostic tests for HCQ retinopathy. Despite these findings, further investigations are needed to establish the potential of ONL thinning as a valuable diagnostic tool for the early detection of HCQ retinopathy. This study thus underscores the importance of continued research in this domain for the advancement of clinical understanding and the development of effective screening strategies.

## Data Availability

The data is available in an Excel sheet, which I can send to you if needed. (Nagib Salameh: nagibsalameh@outlook.com).
